# Changing radiotherapy review practice in response to COVID-19 in a radiotherapy satellite centre

**DOI:** 10.1017/S1460396920000527

**Published:** 2020-06-17

**Authors:** Tracy Mason, Rachael Bennett, Alison White, Agata Rembielak

**Affiliations:** 1 The Christie at Oldham, The Royal Oldham Hospital, Oldham, UK; 2 Clinical Oncology Department, The Christie NHS Foundation Trust, Manchester, UK; 3 School of Medical Sciences, The University of Manchester, Manchester, UK

**Keywords:** radiotherapy review, COVID-19, radiotherapy department, radiotherapy satellite centre

## Abstract

**Background:**

The COVID-19 pandemic has required a rapid and evolving response in order to enable the continued delivery of radiotherapy, whilst effectively managing the risk of infection to patients and staff. In early March 2020, we were presented with the challenge of achieving safe delivery of care in clinical practice for a group of patients that are already at higher risk of morbidity from COVID-19 with only limited guidance.

**Purpose:**

This article outlines the adjustments made at The Christie at Oldham satellite centre in order to promote optimal care to our patients receiving radiotherapy, and to mitigate infection risk at the department for both patients and staff.

**Materials and Methods:**

We share insight into some of the evolving processes that our team have developed since March 2020 in promoting optimal care to patients receiving radiotherapy at The Christie at Oldham satellite centre. These include changes to the department floor, paper flow, supportive care and staffing.

**Results and Conclusions:**

As we continue to progress through this pandemic, we accept that there will be an ongoing journey of learning with adjustments to practice and evolving ‘new norm’, thereby ensuring we keep our patients and team safe and well. We also hoped that our experience would support radiotherapy-related practices in surges of the pandemic in other parts of the world or in case of the second wave.

## Introduction

The COVID-19 pandemic has undoubtedly necessitated healthcare systems worldwide to rapidly react, respond and transform healthcare services in order to mitigate the risk of infection and this has been no less within the speciality of radiotherapy. Some cancer patients are considered to be at a similar higher risk of COVID-19 and possible death, as those with other co-morbidities.^1^ While there are guidelines regarding the change in radiotherapy protocols especially in relation to hypofractionation^2^ and some broader guidelines issued,^3^ what had been lacking is practical guidance as to how to deliver safe care to patients within the clinical setting.

The Christie at Oldham is one of two satellite radiotherapy centres for The Christie NHS Foundation Trust Manchester, UK which is based on site of the Royal Oldham Hospital (as part of The Northern Care Alliance NHS Group). The centre has a pre-treatment suite with a CT scanner and two linear accelerators. The Oldham centre delivers approximately 32,000 fractions of radical and palliative radiotherapy per year. The department team includes treatment radiographers, clinical support workers, engineers, medical physicists and a medical review team. This team consists of two nurse clinicians: a treatment review radiographer and a clinical oncology consultant.

This article will share insight into some of the evolving processes that our team have developed since March 2020 in promoting optimal care to patients receiving radiotherapy at The Christie at Oldham satellite centre. Certainly a fast reaction, teamwork, alongside a problem solving approach were essential in developing risk mitigating approaches to practice in the early days at the start of the pandemic. Practical approaches have been implemented in addition to other Trust-based changes to the delivery of radiotherapy including the implementation of hypofractionated protocols, deferral of radiotherapy priority level 5 treatments,^2^ extension of appointment times in order to manage capacity with the potential for reduced staffing levels and allow for deep cleaning between patients treatments in accordance with strict infection control requirements.

## Changes in Daily Clinical Practice

The changes in practices have evolved rapidly since early March 2020. From a general department perspective we have implemented the following changes:

### Department floor

- Prompt adoption of use of personal protective equipment (PPE) by staff in line with available guidance.^4^ Rapid assessment and requisition for departmental PPE requirements was implemented.
- Designated areas for staff for putting on and removal of PPE in areas as recommended by national guidance.^4^
- Clear local pathways developed for suspected COVID-19 patients detailing the process for moving them to a dedicated isolated area with minimal contact with other patients, for further assessment.
- All general departmental areas are deep cleaned with a chlorine based solution three times a day (Actichlor™ Plus, Ecolab).
- Development of a designated COVID-19 proforma to support telephone triage of patients telephoning with possible symptoms.
- Patients have been advised to phone the department instead of attending if feeling unwell, have symptoms of or had contact with anyone who has symptoms/confirmed COVID-19. They then receive a call back from the medical review team who will undertake more in depth screening.
- Initially, it was implemented that any patients of concern for COVID-19 will be deferred and advised to self-isolate for 7 days. However, at the time of writing this paper, testing for this patient group is now available, enabling those patients who do not test as positive, to resume treatment thereby limiting treatment gaps.
- Patients who themselves are asymptomatic but need to self-isolate due to contact with someone who is symptomatic or confirmed COVID-19, are treated at the end of the day. At that time, the department has been cleared with the exception of minimum staff required for treatment to be safely delivered.
- For all staff and patient/carer, screening at the front door was introduced. It includes a temperature check and key questions, that is, fever >37.8 °C, persistent cough, contact with anyone suspected or confirmed COVID-19 and more recently added if they have experienced a loss of taste and/or smell. Anyone entering the unit is requested to use hand sanitiser to clean their hands before entering and on exit.
- All staff in hospitals in England are expected to wear face masks from 15 June 2020 and all visitors and outpatients must wear face coverings at all times when in the department.
- To minimise footfall through the department, only one entrance is used and the patients are encouraged to attend alone, whenever possible.
- Patients’ relatives or carers are discouraged from attending with the patient for their radiotherapy appointment unless there is a clinical need for them to be present.
- Patient waiting areas have been re-arranged to ensure social distancing and appointment times were adjusted to reduce number of patients in the department.
- There has been a review of patient scheduling and so the department treats at the most risk patient groups (e.g. with lung, elderly, with significant co-morbidities) in the morning on both linear accelerators.
- All items such as patient reading materials that are usually available in waiting areas have been removed.

### Paper flow and supportive care

- Patient paper notes are no longer in use and we utilise the patient’s electronic record with the exception of consent forms that are still required to be signed by the patient. The Trust has also recently allowed for consent to be done by phone. Such consent needs later to be confirmed in writing when the patient attends for radiotherapy.
- Patients who travel to the department by hospital transport are being kept in the radiotherapy department and are not transferred to the discharge lounge which is located in the adjoining acute trust to reduce risk of additional exposure.
- Outpatient prescriptions are taken to pharmacy in batches, delivered to the radiotherapy department and then collected by the patient the following day when they attend for treatment, unless this is an urgent prescription in which case a member of staff will collect this.
- Clarification of practice guidelines concerning resuscitation.^5^ In accepting that there are individuals that are asymptomatic of COVID-19, the hospital Trust decided that all patients should be treated as suspected COVID-19 in the event of a cardiac arrest situation. Initial responders wearing level 2 PPE will therefore only put a defibrillator on the individual and deliver a shock if advised after raising the alert. Full airway and breathing management is not instigated until the cardiac arrest team is present, wearing level 3 PPE.
- Use of an IT platform and telephone interpreter services to support patient contact, where the use of an interpreter is required (especially around consent).
- We have also quickly recognised that patients and their family members or carers have unsurprisingly experienced increased levels of anxiety during the current pandemic. Supportive care, explanation and information have been provided if and when required by telephone or where necessary face to face. The same support is available for the staff.

### Staff and other considerations

- Staff areas now promote social distancing with limitations on numbers of staff in smaller rooms.
- Education events for external delegates and meetings have been cancelled and no external visitors are allowed to visit the department.
- In house staff training continues but this is undertaken with social distancing measures.
- Use of platforms such as MS Teams for meetings and teaching.
- Rapid deployment of IT equipment to enable use of such platforms both with staff and patients/carers.

## Changes to Radiotherapy Review Practice

Traditionally, patients are usually seen by the medical team on a weekly basis during their radiotherapy, in order to proactively assess and manage toxicity. Our practice relating to this has also been changed in order to minimise the time patients are in the department. We decided that factors that impact the decision making process, whether telephone or face-to-face assessment is most appropriate, include:

- Treatment site, dose and likelihood of toxicity.
- Language or other communications issues.
- Urgency of review, for example, for urgent pain management review.
- Patients becoming clinically unwell in the department and requiring urgent assessment.
- Patients requiring consent for treatment if consent by phone is not a viable option.

We now contact patients who are receiving radiotherapy for breast or prostate cancer by telephone. The treating radiographer team grades and document the patient’s radiotherapy related toxicity, for example, skin reaction towards the end of treatment. Some patient groups, including those with lung or head and neck cancers, receive a combination of face-to-face and telephone contacts as it is considered that these groups are at higher risk of side effect and more often require prescription of medicines for management of radiotherapy-related toxicities. We however consider there is still the scope to individualise review plans if patients are coping well with radiotherapy and undertake a telephone consultation instead if this is preferred by the patient. The team is currently piloting a videoconferencing platform (Attend Anywhere®). It is hoped that the platform will facilitate remote face-to-face assessments with patients while at home. Although the use of telephone and videoconferencing has been highlighted to be helpful in avoiding attendance at appointments,^6^ we are currently utilising it to reduce the time patients are required to spend in the department having direct face-to-face contact with different staff, especially where patients have manageable side effects or problems.

Where non-English speaking patients require consent, this is undertaken using our departmental bilingual staff or we use an external translator service and a platform, such as MS Teams. As mentioned earlier, the Trust is moving to a process of consent by telephone for those patients who have been previously seen by their consultant teams and have capacity to consent. When the patient then attends for their treatment, they will then be asked to sign the respective consent form and the name of the clinician who took the telephone consent added to the form.

To date, the Team at the Christie at Oldham have had no staff member tested positive for COVID-19. Although prior to testing being in place, a few staff members had possible symptoms and one most probably had the infection. This could be considered as a reflection of how hard the Team have worked to mitigate the risk for themselves and for our patients. Some of the COVID-19 practices discussed within this article have been adopted throughout The Christie Trust as a whole, while others remain just local to The Christie at Oldham satellite centre. As we continue to progress through this pandemic, we accept that there will be an ongoing journey of learning with adjustments to practice and evolving ‘new norm’, thereby ensuring we keep our patients and team safe and well. We also hoped that our experience would support radiotherapy-related practices in surges of the pandemic in other parts of the world or in case of the second wave.
